# Investigating Machine Learning Techniques for Predicting the Process Characteristics of Stencil Printing

**DOI:** 10.3390/ma15144734

**Published:** 2022-07-06

**Authors:** Péter Martinek, Balázs Illés, Norocel Codreanu, Oliver Krammer

**Affiliations:** 1 Department of Electronics Technology, Faculty of Electrical Engineering and Informatics, Budapest University of Technology and Economics, Műegyetem rkp. 3., H-1111 Budapest, Hungary; martinek.peter@vik.bme.hu (P.M.); illes.balazs@vik.bme.hu (B.I.); 2 Electronic Technology and Reliability Department, Politehnica University of Bucharest, 060042 Bucharest, Romania; norocel.codreanu@cetti.ro

**Keywords:** stencil printing, machine learning, artificial neural network, regression trees, hyperparameter optimization

## Abstract

Stencil printing is the most crucial process in reflow soldering for the mass assembly of electronic circuits. This paper investigates different machine learning-based methods to predict the essential process characteristics of stencil printing: the area, thickness, and volume of deposited solder paste. The training dataset was obtained experimentally by varying the printing speed (from 20 to 120 mm/s), the size (area ratio from 0.35 to 1.7) of stencil apertures, and the particle size (characterized by a log-normal distribution) in the solder paste. Various machine learning-based methods were assessed; ANFIS–adaptive neuro-fuzzy inference systems; ANN artificial neural networks (with different learning methods); boosted trees, regression trees, SVM–support vector machines. Each method was optimized and fine-tuned with hyperparameter optimization, and the overfitting phenomenon was also prevented with cross-validation. The regression tree was the best performing approach for modelling the stencil printing, while ANN with the Bayesian regularization learning method was only slightly worse. The presented methodology for fine-tuning, parameter optimization, and the comparison of different machine learning-based methods can easily be adapted to any application field in electronics manufacturing.

## 1. Introduction

Nowadays, the mass production of electronic devices is dominated by automated surface-mount technology, where electronic components are attached to the printed circuit board by the so-called reflow soldering [1]. This technology consists of applying solder material (increasingly including lead-free alloy since 2006) in the form of a solder paste (a suspension of flux and solder particles) to the contact surfaces of the assembly board, placing the components into the solder paste, and finally transporting the assembly through a conveyor-type oven [2]. In the oven, the entire assembly is heated to above the melting point of the solder. The solder melts everywhere, and after cooling and solidification, the soldered joints are formed. The solder paste is typically deposited by stencil printing, where a printing squeegee is transported across the stencil surface at a specified speed and with a specified force [3]. This causes the solder paste to move, rolling in front of the squeegee and filling the apertures in the stencil above the soldering surfaces. After separating the stencil and the substrate, the solder paste remains on the contact surfaces of the assembly board. In modern circuits, component sizes are continuously reduced to meet the circuit parameters/specifications for wearable, IoT (Internet of Things), and 5G devices. The decreasing size of components (e.g., 200 × 100 µm for passive components) poses a challenge in the most critical step of reflow soldering: the stencil printing for solder paste deposition [4,5].

Research has shown that up to 50–60% of manufacturing defects can be attributed to the stencil printing process [6]; such defects include open joints, due to insufficient solder volume or solder bridges, or short circuits between component leads, due to excessive amounts of the solder. The printing process has become even more critical with the widespread implementation of ultra-fine pitch components such as QFN (Quad-Flat-No-Lead) and µBGA (Micro Ball Grid Array) [7] packages, because these components require even smaller apertures on the stencil than passive components. To improve and accurately control the stencil printing process and achieve zero-defect manufacturing, it is essential to investigate new optimization approaches utilizing machine learning techniques in the case of stencil printing.

Many approaches have been utilized to analyze and enhance the process of stencil printing. As early as 1994, Edwards showed that early-phase process optimization (at the product or production planning stage) significantly increases process goodness, the so-called “first-pass yield” (the ratio of defect-free prints to defective prints), and reduces the defect rate already at the stage of the process setup [8]. This means that the process setup can be faster, mass production can start sooner, and errors during production can be kept to a minimum.

In the past, empirical optimization methods were used to improve the stencil printing process, e.g., Taguchi or DMAIC (Define, Measure, Analyze, Improve, and Control). Later, methods based on numerical modelling and, more recently, machine learning became more widespread. For stencil printing, Li et al. performed a DMAIC-based study [9]. They analyzed which process and environmental parameters have the most influence on the deviation of the applied solder paste height. The most influencing parameters included the size of the stencil aperture, the printing speed, and the type of the solder paste (defined by the particle size in the paste). Tsai also investigated the stencil printing process and improved it using RSM (response surface methodology) [6]. They found that the quality of the solder paste deposition at fine-pitch components depends to a large extent on the thickness of the stencil. The thicker the stencil foil, the less the solder paste will be transferred to the substrate at small apertures [6]. That is why the size of the apertures is typically characterized by their area ratio (1) [10], which takes both the linear dimensions of the aperture and the thickness of the stencil into consideration.

(1) $$AR=\frac{w\cdot l}{2\left(w+l\right)t}$$

where *AR* is the area ratio of the aperture, *w* and *l* are the linear dimensions of the stencil aperture, and *t* is the thickness of the stencil foil.

In the last decade, the available computational resources have increased significantly, allowing the application of computationally intensive methods to analyze and estimate the parameters of non-linear processes. Optimizations applied in the design phase of electronics manufacturing processes can significantly increase the first pass yield, reducing the number of repairs and re-soldering [11].

Since the 2000s, the stencil printing process has been studied using machine learning methods. Tsai trained an artificial neural network to study the effect of different printing parameters on the print quality [6]. The input parameters included squeegee force, printing speed, stencil foil thickness, solder paste viscosity, solder paste type, and aperture size for components with different lead pitches. The output parameter space consisted of two elements: the volume of deposited solder paste and a value derived from a subjective judgement of quality. Finally, the quality of the stencil printing was characterized by a so-called desirability value (*D*), which was derived from the output parameter space by the following relation (2) [6]:

(2) $$\begin{array}{l} d_{i}=\left\{\begin{array}{l} \left|\frac{\mu_{i}-{\overset{⌢}{y}}_{i}}{SL_{i}\cdot \mu_{i}}\right|,\;\left(1-SL_{i}\right)\cdot \mu_{i}\leq {\overset{⌢}{y}}_{i}\leq \left(1-SL_{i}\right)\cdot \mu_{i}\\ 1,\;\mathrm{otherwise} \end{array}\right.\\ D={\left(\prod_{i=1}^{n}d_{i}\right)}^{\frac{1}{n}} \end{array}$$

where *d_i_* is the acceptability function of the *i*-th response parameter, *y* is the estimator of the *i*-th parameter, *µ_i_* is the target value of the *i*-th parameter, *SL_i_* is the specification limit of the *i*-th parameter (20–25% for most parameters in stencil printing based on industry standards), *D* is the acceptability function of the whole system, and *n* is the length of the response vector (number of response parameters). Note that the authors suggest calculating the geometric mean of (1 − *d_i_*) for *D*, because if the estimator of one of the response parameters (*y*) equals the target value (*µ*), then *d_i_* will be zero, rendering the value of *D* to zero (which is the ideal value by the equation in [6]) for the whole system.

Using a neural network, Tsai could estimate the volume of the deposited solder paste and the print quality with a relative error of about 5–6%, while the acceptability function was estimated with an error of about 8%. The shortcomings of Tsai’s study are that the aperture size was only investigated for QFP components with three different pitch sizes, and the viscosity of the solder paste was only characterized by a single numerical value. Solder pastes are non-Newtonian liquids, meaning their viscosity depends on the shear rate. Yang and Tsai developed a neuro-fuzzy control system for optimizing stencil printing [12]. In their study, the training vector was the same as that in their previously presented work (squeegee force, printing speed, stencil thickness, etc.). To characterize the estimation performance of their method, they used the so-called percentage RMSE (Root Mean Square Error). They estimated the volume and height of the deposited solder paste with an error of 3% and 1–2%, respectively. Tsai and Liukkonen [13] investigated the stencil printing process using fuzzy logic and an artificial neural network. In the first method, fuzzy logic was used to determine the output values. Then, the Taguchi method was used to give the optimal combination of parameters for a given target value of the output parameters. In the second method, an artificial neural network was trained on experimental data, and a genetic algorithm was used to find the optimal combination of parameters. The hybrid approach based on fuzzy logic and the Taguchi method proposed by the authors proved to be the most efficient, with an average of 23% improvement in the estimation of deposited solder paste volume.

In this paper, we aimed to fill the gaps identified in the literature and investigate the description of the stencil printing process using different machine learning methods (neuro-fuzzy inference systems, artificial neural networks, regression trees, boosted tree and support vector machines), taking into consideration the particle size in the solder paste and the size (described by their area ratio) of the stencil apertures.

## 2. Materials and Methods

### 2.1. Experiment for Acquiring the Training Dataset

Our study investigated the stencil printing process using machine learning-based methods. The most critical step in machine learning is constructing the training set. It should include input process parameters that are significant for stencil printing and the output parameter space that characterizes the quality of stencil printing, the most important of which is the so-called printing efficiency. Printing efficiency is the ratio of solder paste volume applied on the printed circuit board surface to the volume of the stencil aperture (3) [14]. Its value should be in the range of 100% ± 20–25% for a good-quality print.

(3) $$TE=\frac{V_{deposited\_solder}}{V_{aperture}}$$

where *TE* is the printing efficiency, *V_deposited_solder_* is the volume of solder paste that has been deposited, and *V_aperture_* is the volume of the stencil aperture.

Printing efficiency essentially depends on four parameters: the machine capability of the stencil printer, which includes all printing parameters (e.g., printing speed); the rheological properties of the solder paste (affected by the particle size range in the paste); the stencil fabrication technology (laser cutting, electroforming, etc.); and the size of the stencil aperture, which is most commonly described by its area ratio (1). The following parameters were considered for the construction of the training set: printing speed (20, 45, 70, 85, 120 mm/s), the rheology of the solder paste using different types of solder pastes, and the aperture size (area ratio from 0.35 to 1.7) The other fixed parameters were the following: specific squeegee force (0.3 N/mm); stencil separation speed (10 mm/s); stainless steel stencil foil with a thickness of 150 μm, on which the apertures were formed by laser cutting. The surface finish of the printed circuit board was immersion silver.

**Figure 1:**
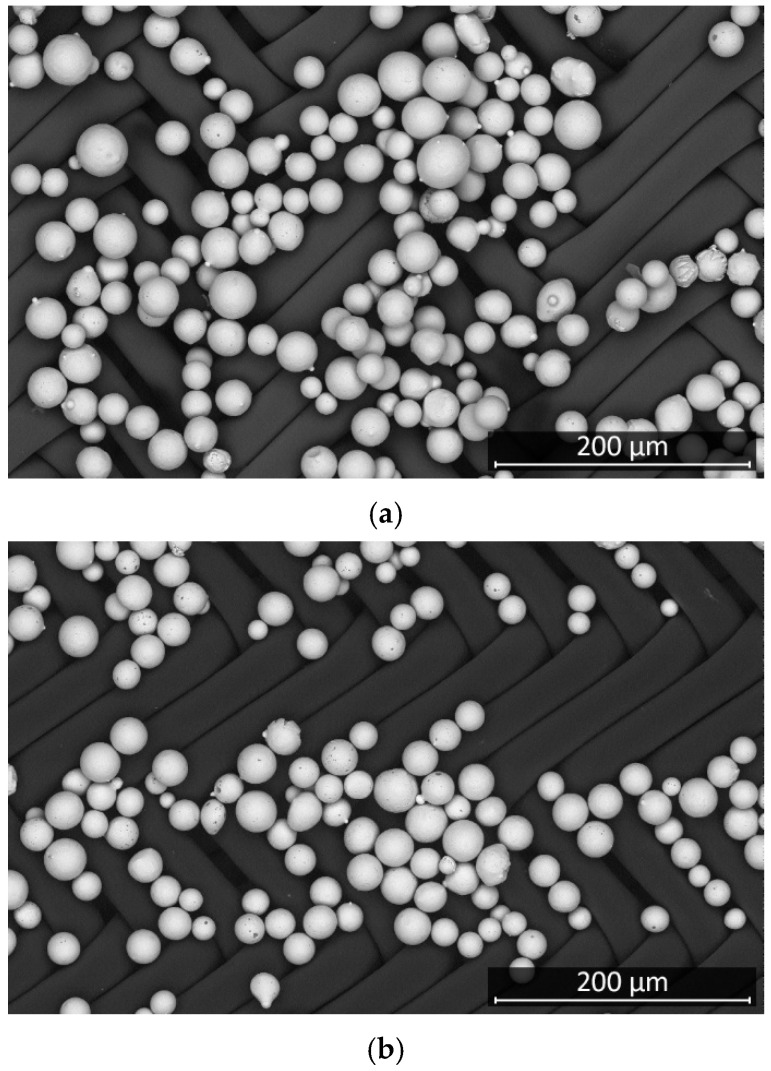

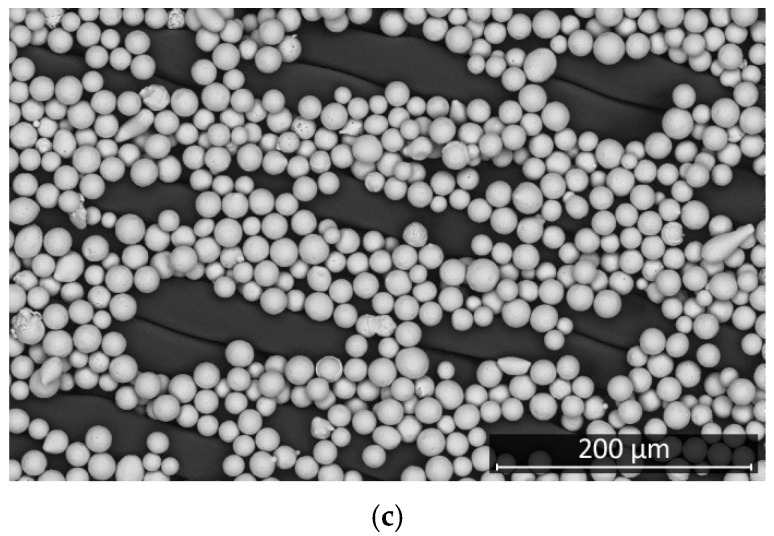
Scanning electron microscopy images of solder paste grains: (**a**) Type-3 solder paste; (**b**) Type-4 solder paste; (**c**) Type-5 solder paste.

The effect of the rheology of the solder pastes was investigated by applying three different types of solder pastes, where the type indicates the range of the particle size of the solder pastes. The flux vehicle system in the pastes was kept the same so as not to affect the study results. The theoretical particle size ranges for each paste are as follows: Type-3: 25–45 μm; Type-4: 20–38 μm; Type-5: 15–25 μm. To characterize the solder pastes quantitatively in our teaching set, the grain size distribution of each solder paste was determined. To this end, scanning electron microscope images were first taken of the particles in the solder pastes (Figure 1), and then the diameter of the particles was measured using an image processing-based method implemented in Matlab 2019b.

**Figure 2:**
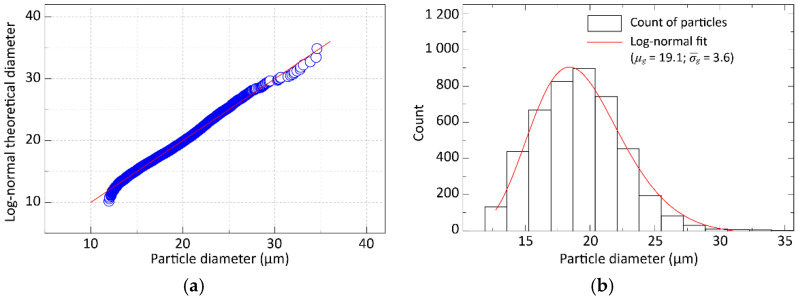
Log-normal distribution fitted to the particle size distribution of Type-5 solder paste: (**a**) Lognormal Q-Q plot of particle diameter; (**b**) Histogram plot of particle size distribution.

We found that the particle size in all types of the investigated solder pastes could be characterized by a log-normal distribution with characteristic parameters of the geometric mean (*μ_g_* = *e^µ^*) and geometric standard deviation (*σ_g_* = *e^σ^*). The fit of the log-normal distribution to the particle size distribution in the Type-5 solder paste is shown in Figure 2.

With these parameters, quantitative input values could be used to teach the machine learning method instead of qualitative parameters such as Type-3, Type-4, and Type-5. The log-normal fitting parameters for different types of solder pastes are given in Table 1.

The output (response) parameters were the volume, height, and area of the solder paste deposits, which were measured using an automated 3D solder paste inspection (SPI) machine as a function of the input parameters. The total experiment consisted of 75 test runs (5 printing speeds, 3 solder paste types, 5 repeats), and the solder paste deposits were inspected at 209 locations on each test board. As a result, the training set contained 15,675 vectors of output and input parameters.

### 2.2. Investigated Machine Learning-Based Methods

Machine learning-based methods perform with different accuracies depending on the given non-linear problem being modelled. The following methods have been investigated for predicting the output of stencil printing: neuro-fuzzy inference systems (ANFIS), artificial neural networks (ANN), regression trees (RegTree), boosted trees (BoostTree), and support vector machines (SVM). Each method has been optimized because a simpler structure may not be able to model the process, resulting in lower accuracy. At the same time, a structure that is too complex can cause overfitting, which means that, in the case of new input parameters, the prediction accuracy can radically drop.

As a structure parameter, the number of the applied membership functions must first be determined for the method of ANFIS. Membership functions are exclusively dedicated to input dimensions; hence, they were divided into four groups based on the four input parameters (printing speed, area ratio of apertures, geometric mean, and geometric deviation of solder paste particle size). The ANFIS systems predict only one output (output with one dimension). Hence, three models were built and optimized to predict the three output values (the area, height, and volume of solder paste deposits). Triangular and Gaussian membership functions were applied by a learning process consisting of several learning iterations. This made it possible to select the type of membership functions, leading to higher accuracy in our model. Accuracy was defined by calculating the mean absolute percentage error (MAPE). The required minimum number of learning iterations was defined next. The learning process with more iterations becomes more accurate, but the learning time increases significantly. That is why a required minimum was calculated, where the accuracy is acceptable and the learning time is not more than an hour, even in the case of more complex structures. The ideal size and configuration of the ANFIS were defined by an iterative grid search method. Working parameters were defined during the learning process, and MAPE values were calculated for all the different outputs (area, height, and volume of paste deposits). While these were calculated for other machine learning-based methods too, the performance of the ANFIS could be easily compared with them (see the detailed results in Section 3).

Both the optimal size and the best learning method were defined during our approach in the case of neural networks. The Levenberg-Marquardt (LM) and the Bayesion Regularization (BR) learning methods were applied and compared for structures of different complexities. The optimal number of neurons was defined next by increasing that gradually and calculating the prediction error. Care was taken to ensure that a structure containing too many neurons can cause overfitting. MAPE values were calculated for all outputs and compared with the results of other machine learning-based methods.

Regression trees provided high accuracy even without the fine-tuning of the minimum leaf size. By introducing cross-validation for the prevention of overfitting, we found that the model with a lower number for the minimum leaf size may cause overfitting; the calculated MAPE was almost zero in our first model. Hyperparameter optimization with cross-validation was then applied to suggest a minimum leaf size with an acceptable error, while simultaneously preventing overfitting. Learning time was also measured and considered during the optimization.

The boosted tree is a hybrid method combining the simplicity of decision trees with robust learning methods. Three model parameters were optimized for boosted trees: the minimum leaf size, the learning rate (which defines the sensitivity of the learning process), and the number of learning iterations. These parameters were determined with hyperparameter optimization first. After calculating the MAPE values, the connections among them and the model parameters were also analyzed in detail. By refining the model parameters (increasing learning iterations, increasing the learning rate, and decreasing the minimum leaf size), the MAPE value reduced significantly, indicating the overfitting of the model. Overfitting could have been prevented by cross-validation iterations, but the accuracy also decreased considerably. The calculated optimal structure parameters and MAPE values for the boosted trees are also presented in Section 3.

The support vector machine can use different kernel functions such as linear, polynomial, or Gaussian. With some test runs, the most suitable was selected by comparing errors on some simpler structure for modelling the stencil printing. Three parameters were then optimized by hyperparameter optimization: the box constraint, epsilon, and the kernel scale. The overfitting was prevented by applying multiple learning iterations with cross-validation for different training datasets.

## 3. Results and Discussion

The chapter presenting the results is divided into two parts: the first part deals with the optimization results of the different prediction methods. The second covers the detailed comparison of the various methods for predicting the output parameters of stencil printing.

### 3.1. Optimization of Prediction Methods

**Figure 3:**
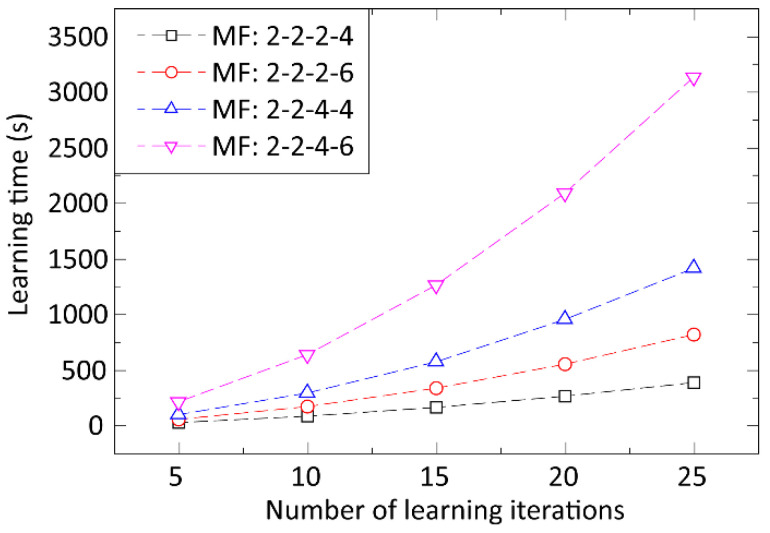
Learning time as a function of the learning iteration number, the configuration, and the total number of membership functions (MF).

**Figure 4:**
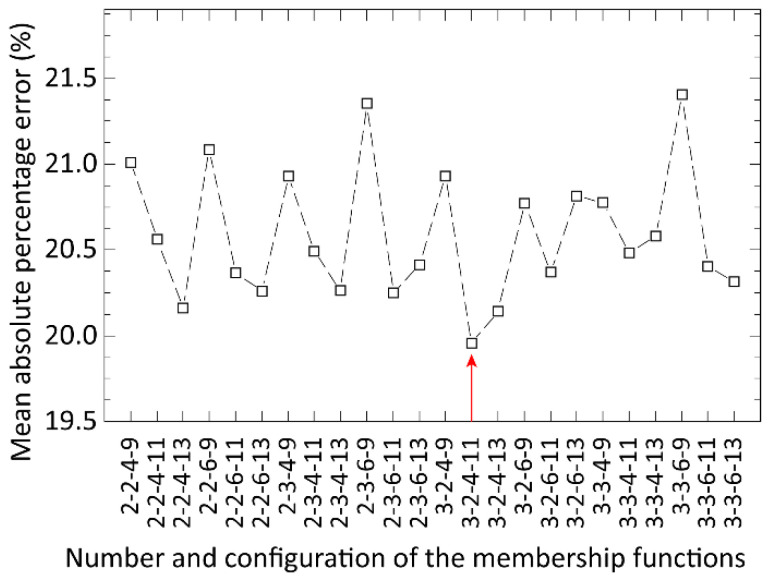
Error of prediction (MAPE) as a function of configuration and the total number of membership functions.

In the optimization of the ANFIS method, the membership functions were divided into four groups and connected exclusively to the four process input parameters. Based on our input data, membership functions should ideally be divided by a ratio of 3:3:7:23 for the input dimensions (geometric mean and geometric deviation of solder paste particle sizes, printing speed, and the area ratio of apertures, respectively). The three models for the three process output parameters (area, height, and volume of solder paste deposits) were built and optimized for the prediction. Triangular and Gaussian types of membership functions were investigated at first by learning processes consisting of 10 learning iterations and a low total number of membership functions, varying from 10 to 14. Using the Gaussian membership function provided a 10–20% lower prediction error, so this membership function was used for further optimizations. Next, the prediction error and the learning time were analyzed as a function of the learning iteration number (utilizing a low total number of membership functions from 10 to 14 for this test). The prediction error was primarily unaffected by the number of learning iterations (varying from 5 to 25), but the learning time increased significantly (Figure 3). Based on this, the number of learning iterations was set to 10 for optimizing the complexity of the ANFIS structure, i.e., the total number and division of membership functions. The analysis is illustrated in Figure 4. The best accuracy was provided by applying an ANFIS structure consisting of 20 membership functions in a distribution of 3:2:4:11 (geometric mean, geometric deviation, printing speed, area ratio). The overall prediction error, averaged by the error (MAPE) for predicting height, area, and the volume of solder paste deposits, was 19.9% for this ANFIS structure. The required learning time by using 10 learning iterations was 89 min in this case.

**Figure 5:**
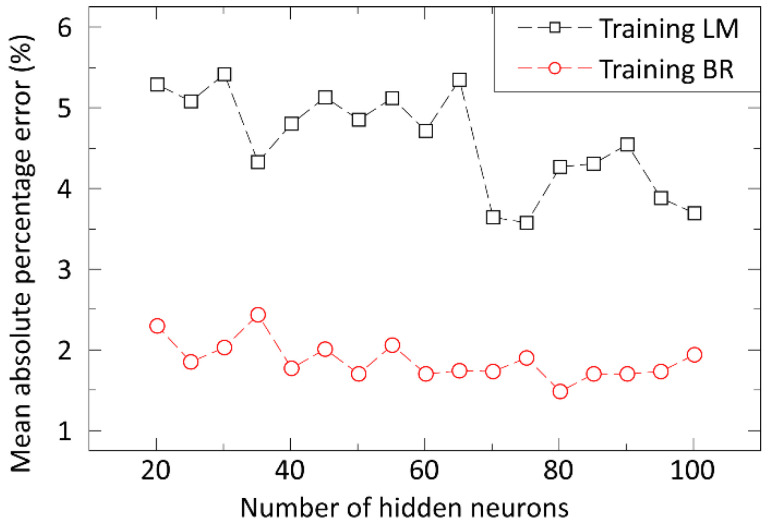
The error of prediction (MAPE) as a function of the number of hidden neurons.

For training the artificial neural network (ANN), the Levenberg–Marquardt (LM) and Bayesian Regularization (BR) learning methods were investigated and compared together using structures of different complexity, i.e., the number of hidden neurons. Figure 5 shows the error of ANNs trained by these methods.

The BR training proved to be more effective in our case. As the number of neurons is generally low (the applied tuning factor is less than 1), no overfitting was detected in our predictions for different input datasets. The optimal number of hidden neurons for modelling the process of stencil printing with a neural network was 80, where the prediction error (MAPE) was 1.5%. The learning time was a few minutes with the BR learning method and less than a minute with the LM method, so both could be trained in a reasonably short time.

**Figure 6:**
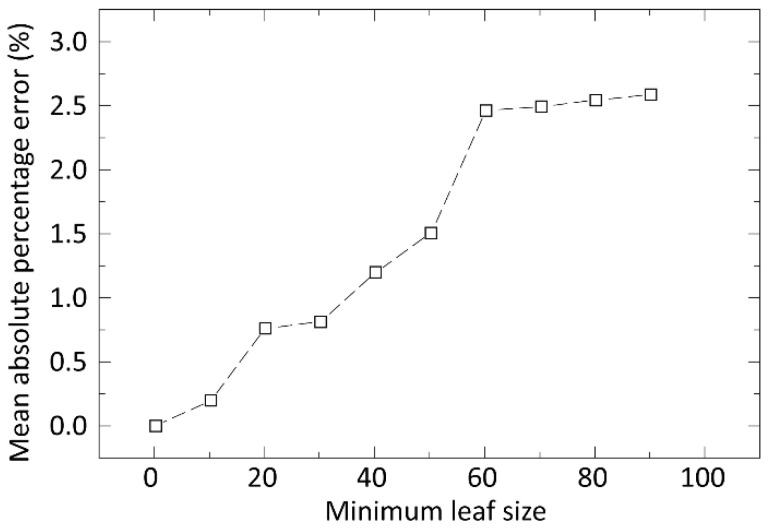
MAPE of prediction based on the parameter minimum leaf size.

Regression trees provided a high accuracy even without any fine-tuning. However, the influence of change in the minimum leaf size parameter was evaluated. Figure 6 illustrates the mean absolute percentage error of prediction based on the minimum leaf size.

By performing cross-validation, it was found that a model with a lower minimum leaf size caused overfitting: the calculated MAPE was 3.83 × 10^−6^ for the model, with a minimum leaf size of 1. The hyperparameter optimization with cross-validation suggested a minimum leaf size of 73, yielding a prediction error of 2.2%. By subsequently applying 15 cross-validation datasets, the MAPE was decreased to 0.8%, eliminating overfitting by setting the minimum leaf size to 3. The learning time was less than a second.

**Figure 7:**
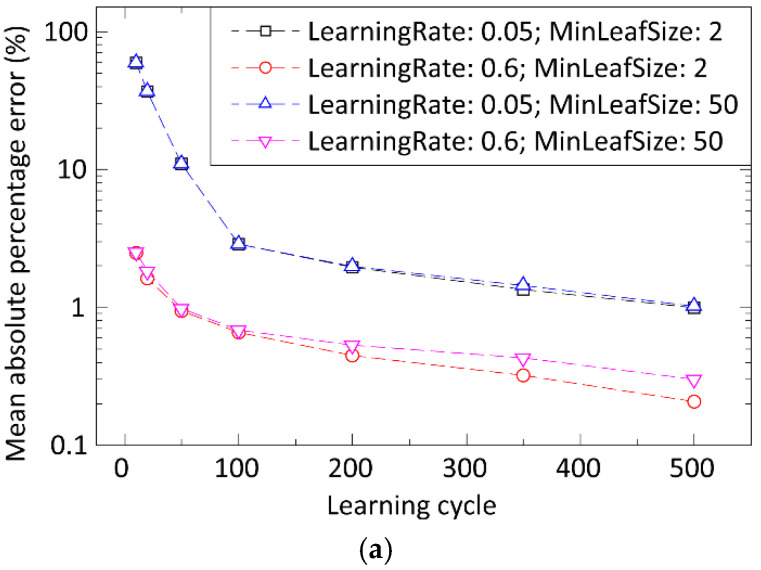

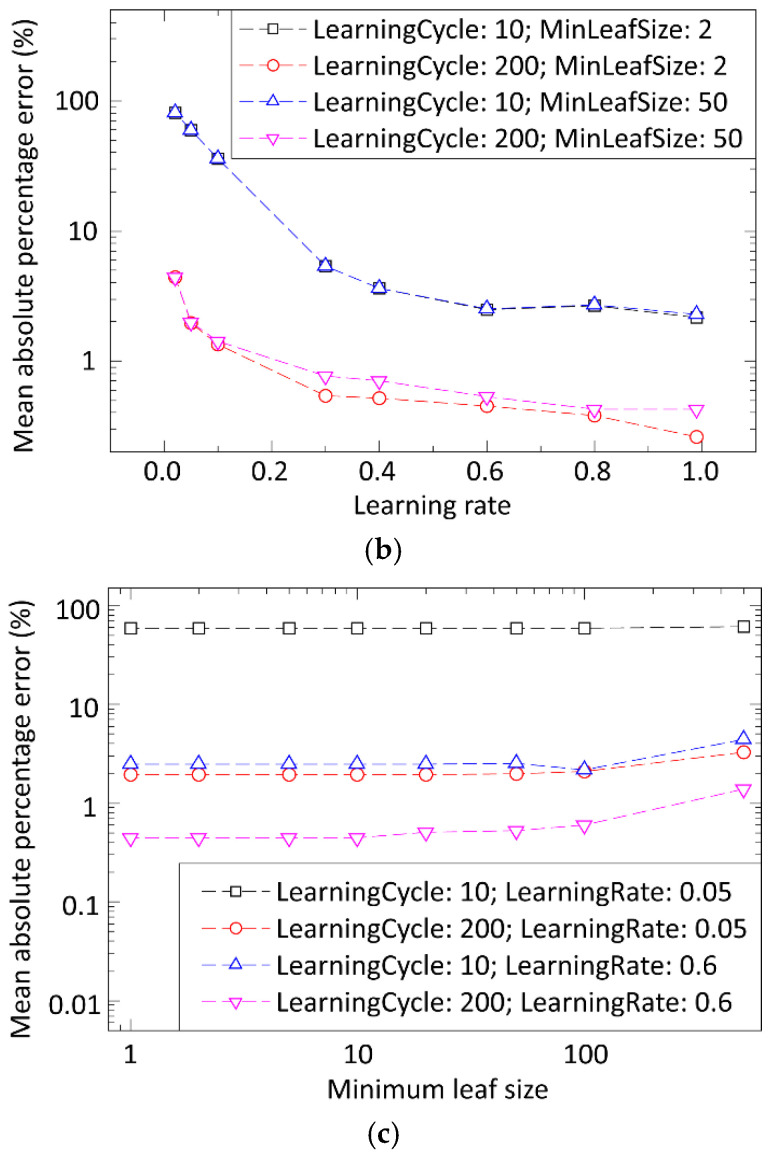
Prediction error of deposited solder paste volume based on the different parameter settings as a function of the: (**a**) number of learning iterations; (**b**) learning rate; (**c**) minimum leaf size.

In the case of Boosted Trees, three parameters were optimized: the minimum leaf size, the learning rate, and the number of learning iterations. The hyperparameter optimization calculated the possible mean leaf size values between 1 and 29, the learning rate between 0.3 and 0.5, and the learning iterations between 11 and 495 for the stencil printing output parameters. Figure 7 illustrates the prediction error for the deposited solder paste volume as a function of these parameters.

It was found that refining the parameters (increasing learning iterations, increasing the learning rate, and decreasing the minimum leaf size) can radically reduce the MAPE value, resulting in overfitting. By applying cross-validation iterations, the optimal parameter settings were: 5 for the minimum leaf size and 0.1 for the learning rate with 30 learning cycles. The MAPE was 5.9%, whereas the learning time was less than a second with these settings.

**Figure 8:**
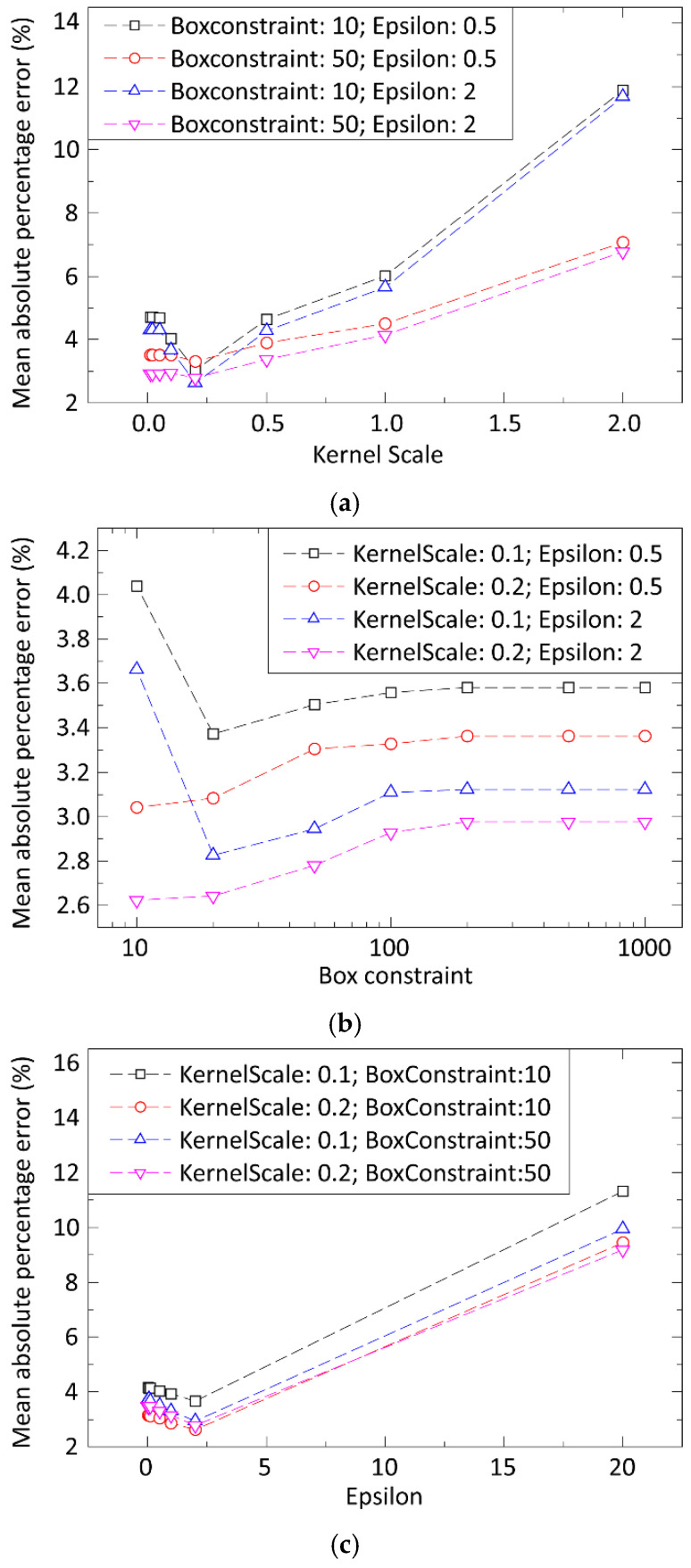
Prediction error of deposited solder paste volume based on the different parameter settings as a function of the: (**a**) Kernel scale, (**b**) Box constraint, (**c**) Epsilon.

In the case of Support Vector Machines, it was found that by using the Gaussian kernel function, the model clearly outperformed other models using different kernel functions. Hence, the Gaussian kernel function was used in the subsequent optimizations. Three parameters (Box constraint, Epsilon, and Kernel scale) were fine-tuned next. Prediction errors as a function of model parameters are illustrated in Figure 8.

Hyperparameter optimization was applied to calculate the optimum model parameter settings, and cross-validation in five iterations was applied to prevent overfitting. The resulting model parameters were 10, 1, and 0.2 for the Box constraint, Epsilon, and Kernel scale, respectively. The prediction error was 5.9% for the average error for the deposited paste volume, height, and area.

### 3.2. Comparing the Prediction Methods

**Figure 9:**
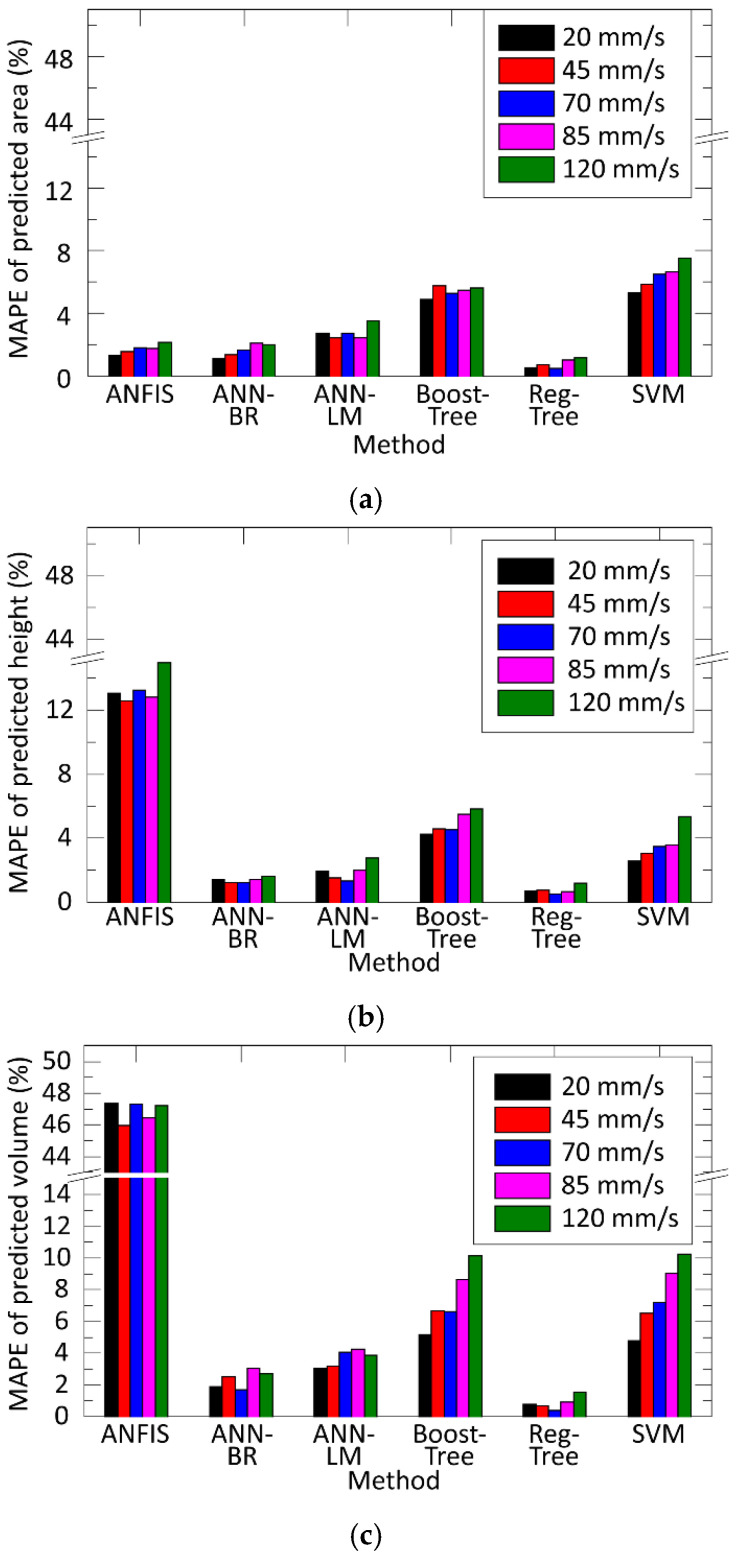
Comparing the prediction error as a function of printing speed: (**a**) error for the printed height; (**b**) error for the printed area; (**c**) error for the deposited paste volume.

Finally, the prediction error of all the methods has been compared together for the different process output parameters (deposited paste area, height, and volume). Figure 9 illustrates the comparison as a function of printing speed. The method of the regression tree was the best but the Artificial Neural Network, applying Bayesian Regularization for the learning (ANN-BR), were just slightly worse.

**Figure 10:**
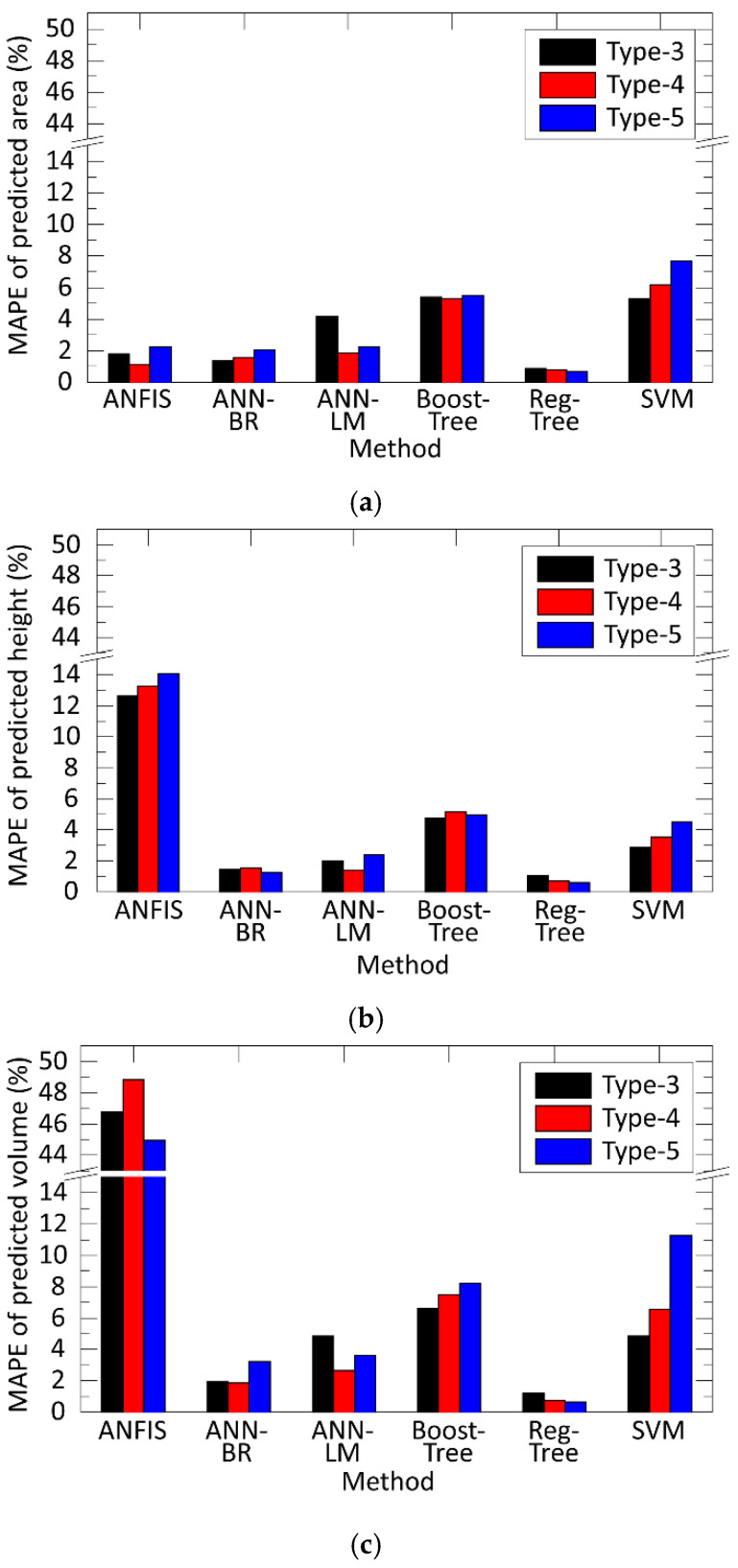
Comparing the prediction error as a function of the paste-type (particle size): (**a**) error for the printed height; (**b**) error for the printed area; (**c**) error for the deposited paste volume.

The same comparison of prediction errors was carried out as a function of paste types, the results of which are illustrated in Figure 10. The results were the same in this case; the regression tree and the artificial neural network with Bayesian Regularization were the methods with the lowest prediction errors. In both cases, ANFIS provided a significant prediction error. Thus, it was not appropriate for the modelling of stencil printing.

## 4. Conclusions

Various machine learning-based methods (neuro-fuzzy inference systems, artificial neural networks, regression trees, boosted tree, and support vector machines) were investigated for predicting the process yield of stencil printing. Machine learning-based methods are simply compared to each other many times in the literature without any parameter optimization and with the risk of overfitting. It was proven in this work that the different machine learning methods could be compared together only after careful model parameter optimization and the elimination of the phenomenon of overfitting. We propose using a regression tree or neural network to predict the output values (area, height, and volume of the deposited solder paste) for the stencil printing process based on the input data (the particle size in the solder paste, the printing speed, and the aspect ratio). The learning time was only a few seconds in the case of the regression tree, so this method can be used even for real-time prediction of the yield of stencil printing. The approach presented in this paper can be a helpful guide to find the most suitable method to solve any kind of non-linear prediction problem.

## Figures and Tables

**Table 1:** Log-normal fit parameters for different types of solder pastes.

Solder Paste Type	Type-3	Type-4	Type-5
Geometric mean (*μ_g_*)	26.6	25	19.1
Geometric deviation (*σ_g_*)	8.1	4.4	3.6

## Data Availability

The raw/processed data required to reproduce these findings cannot be shared at this time, as the data also form part of an ongoing study.
